# Characterizaton of the Vessel Geometry, Flow Mechanics and Wall Shear Stress in the Great Arteries of Wildtype Prenatal Mouse

**DOI:** 10.1371/journal.pone.0086878

**Published:** 2014-01-27

**Authors:** Choon Hwai Yap, Xiaoqin Liu, Kerem Pekkan

**Affiliations:** 1 Department of Biomedical Engineering, National University of Singapore, Singapore, Singapore; 2 Department of Developmental Biology, University of Pittsburgh School of Medicine, Pittsburgh, Pennsylvania, United States of America; 3 Department of Biomedical Engineering, Carnegie Mellon University, Pittsburgh, Pennsylvania, United States of America; Centrum Wiskunde & Informatica (CWI) & Netherlands Institute for Systems Biology, Netherlands

## Abstract

**Introduction:**

Abnormal fluid mechanical environment in the pre-natal cardiovascular system is hypothesized to play a significant role in causing structural heart malformations. It is thus important to improve our understanding of the prenatal cardiovascular fluid mechanical environment at multiple developmental time-points and vascular morphologies. We present such a study on fetal great arteries on the wildtype mouse from embryonic day 14.5 (E14.5) to near-term (E18.5).

**Methods:**

Ultrasound bio-microscopy (UBM) was used to measure blood velocity of the great arteries. Subsequently, specimens were cryo-embedded and sectioned using episcopic fluorescent image capture (EFIC) to obtain high-resolution 2D serial image stacks, which were used for 3D reconstructions and quantitative measurement of great artery and aortic arch dimensions. EFIC and UBM data were input into subject-specific computational fluid dynamics (CFD) for modeling hemodynamics.

**Results:**

In normal mouse fetuses between E14.5–18.5, ultrasound imaging showed gradual but statistically significant increase in blood velocity in the aorta, pulmonary trunk (with the *ductus arteriosus*), and descending aorta. Measurement by EFIC imaging displayed a similar increase in cross sectional area of these vessels. However, CFD modeling showed great artery average wall shear stress and wall shear rate remain relatively constant with age and with vessel size, indicating that hemodynamic shear had a relative constancy over gestational period considered here.

**Conclusion:**

Our EFIC-UBM-CFD method allowed reasonably detailed characterization of fetal mouse vascular geometry and fluid mechanics. Our results suggest that a homeostatic mechanism for restoring vascular wall shear magnitudes may exist during normal embryonic development. We speculate that this mechanism regulates the growth of the great vessels.

## Introduction

Congenital cardiovascular malformations (CCM) affect 0.6%–1.9% of live births [1], and due to its complexity, its developmental etiology is not completely understood. Challenges in imaging techniques exacerbate the problem. Altered fluid mechanical environment in the fetal cardiovascular system has been hypothesized to play a role in causing CCM. This stems from evidence where altered mechanical force environment were observed to cause altered phenotypes in animal models [2]–[4]. For example, Tobita et al. [4] showed that banding the left side of the common atria in chick embryos can result in hypoplastic left heart phenotypes; while Hogers et al. [3] showed that uni-lateral ligation of chick vitelline vein can cause septal defects and pharyngeal aortic arches defects.

Experiments in biology have provided further mechanistic evidence to these observations. Unique studies showed that altered embryonic hemodynamics can alter expression of shear-sensitive proteins such as Kruppel-like factor 2 (KLF2), endothelial nitric oxide synthase (eNOS), endothelin-1 (ET1) in the early embryonic outflow tract and aortic arteries [5]. Likewise the hemodynamic shear stress can modulate signaling pathways involving extracellular regulated kinases-1/2 (ERK1/2), nuclear transcription factor-κB (NF-κB), bone morphogenetic protein-4 (BMP-4), vascular endothelial growth factor (VEG-F) and NOTCH [5]–[8]. Further, recent work showed the presence of primary cilia on the endothelium of fetal mouse aortas [9], and it was proposed that these cilia function as vascular mechanosensors for wall shear stress.

Given the potential importance of hemodynamic mechanical stimuli for proper cardiovascular development and in congenital malformations, as suggested by the above studies, we proposed that correcting the mechanical force environment *in utero* may prevent, alleviate, or perhaps even rescue certain types of CCM. *In utero* interventions may also be contemplated in preventing aortic arch interruption and coarctation, as it has been proposed these two CCM templates may also arise from reduced flow in the aorta [10]. *In utero* interventions may be particularly advantageous given the potentially greater regenerative capacity and plasticity of the developing vasculature, as demonstrated by a recent study that showed that 1-day-old neonatal mouse has enhanced regenerative response to cardiac wounds, but this capacity is lost by 7 days of age [11]. In fact, some investigators have attempted *in utero* balloon valvuloplasty, and reported that they averted the formation of single ventricle malformations [12], [13]. As *in utero* fetal cardiovascular intervention continues to advance, a good understanding of fetal cardiovascular hemodynamics and how it evolves over development can help guide surgical interventions to recover function. A good understanding of fetal cardiovascular mechanobiology will also aid the same effort, and mechanobiology studies require the characterization of native mechanics as a pre-requisite.

In the current paper, we characterize the prenatal fluid mechanical forces in the developing aorta, using the wildtype mouse as the animal model. We employed a novel method that combines three different techniques: ultrasound biomicroscopy (UBM), cryo-episcopic fluorescence image capture (EFIC) and computational fluid dynamics (CFD).

## Methods

### Mouse Models and Ethics Statement

To harvest fetal mouse pups, the pregnant mother mouse was first euthanized in the CO_2_ chamber, and her womb was recovered. Fetuses were then collected through careful dissection under the microscope. The number of fetuses investigated is as follows: 3 embryos at E14.5, 4 embryos at E15.5, 5 embryos at E16.5, 4 fetuses at E17.5, and 3 fetuses at E18.5. All animal studies were approved by the University of Pittsburgh Institute Animal Care and Use Committee (Protocol number 12070410).

### Ultrasound Biomicroscopy

Ultrasound imaging was used to measure flow boundary conditions for use in CFD simulations. Imaging was performed using the Vevo Imaging Station (Visual Sonics Inc., Toronto, Canada) which comes with heating pad, rectal thermometer, ECG, adjustable stage and transducer holders. The adult pregnant mother mouse being imaged was sedated with isoflurane gas (initially at 4%, then maintained at 1.5%), and placed on a heating pad where imaging was done. Thoracic hair was removed with depilatory cream, and the pre-warmed ultrasound gel was applied before imaging. Body temperature was tracked with a rectal probe and heart rate was tracked with ECG leads on the heating pad. To minimize disturbance to mother and fetal cardiovascular system due to anesthesia, body temperature was maintained at 36±0.5°C (additional heating was provided with a heating lamp from above the mouse when necessary), and heart rate at 400–550 beats/min. Measurements of pulsatile velocity waveforms were carried out with pulse-wave (PW) Doppler echocardiography Vevo 2100 UBM (Visualsonics Inc., Toronto, Canada) with a 40 MHz transducer. Measurements were performed in the aortic and pulmonary roots, the thoracic descending aorta (at diaphragm level), the innominate artery and the left common carotid artery, with the angle between the ultrasound beam and the direction of flow being less than 30°. Methods for these measurements were published previously [14], [15].

### Episcopic Fluorescence Image Capture (EFIC)

**Figure 1:**
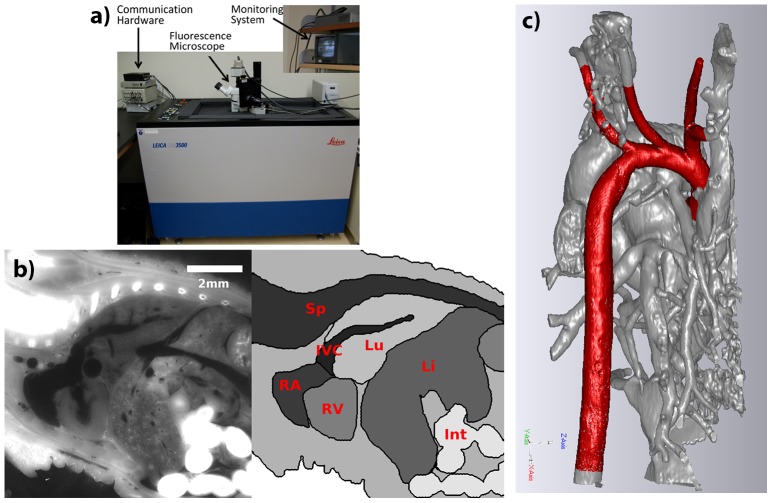
Imaging cardiovascular geometries with cryo-EFIC. a) The cryo-EFIC machine and its components. b) Sample EFIC image slice of a newborn mouse, demonstrating that there were contrasts between different tissues to distinguish the organs. Int - intestines; c) Demonstration of segmentation: rendered image of the blood volumes (gray) of a newborn mouse, with the aorta and its head and neck arterial branches set apart in red. d) Segmented great arterial geometries of wildtype fetal mice over stages E14.5 to E18.5 compared to that of the newborn mouse. IVC – inferior vena cavae; Li – liver; Lu – lungs, RA - right ventricle; RV – right ventricle; Sp – spine; AAo: ascending aorta; DAo: descending aorta; PT: pulmonary trunk; Duc: ductus arteriosus; Innom: innominate; LCC: left common carotid; LSC: left subclavian.

EFIC [16], [17] was employed to obtain detailed 3D fetal organ geometries for our mechanics study. The cryo-embedding version of EFIC imaging was employed (Figure 1a) instead of the paraffin-embedding version because in the latter, tissue shrinkage occurs due to dehydration and distorted native geometries. The EFIC machine consisted of a sledge cryo-microtome (CM3500 Cryopolycut, Leica Microsystems GmbH., Germany) custom attached to a mercury lamp fluorescence microscope (MZ16 FA Fluorescent Stereomicroscope, Leica Microsystems GmbH., Germany). Whole fetus pups were immersed in cryo-embedding matrix (M1 embedding matrix, Thermo Fisher Scientific Inc., Waltham, MA, or Tissue-Tek® OCT Compound, Sakura Finetek, Japan) and snap-frozen using liquid nitrogen immediately after harvesting. The frozen blocks were then sectioned at 2–5 micron thickness in the cyo-microtome, and the blocks were imaged with the microscope with a GFP emission filter after every slice using tissue auto-fluorescence excited by a 475 nm wavelength light source. The entire EFIC process was automated with custom-built electronics and by a custom-written OpenLab® (Perkin-Elmer, Waltham, MA) program.

### Analysis of Geometric Parameters

EFIC imaging can have both in-plane and out-of-plane resolution as fine as 2 microns. The 2D serial EFIC image stacks were processed with MATLAB® (Mathworks Inc., Natick, MA) for cropping, down-sampling and contrast enhancement. An Anisotropic Diffusion Speckle Filter algorithm [18], [19] was applied to smooth out localized noise. 3D blood volumes in major vessel were segmented with an open source program, VMTK [20], which was also used to calculate vessel centerlines and geometric features. Calculation methods employed by VMTK are published previously [21], [22]. For geometric feature calculations, the aortas were clipped of all branches using VMTK, and the resulting hole was filled using the curvature-based hole filling algorithm in Geomagic Studio® (Geomagic Inc., Morrisville, NC).

### Computational Fluid Dynamics (CFD)

Smoothing and boundary extension were applied on great arterial geometries from VMTK with Geomagic Studio®. All boundaries were extended by at least 9 diameter lengths, except for the pulmonary and aortic roots, which were only given 1 diameter extension due totheir proximity to the ventricles. The geometry was meshed using Gambit® (Ansys Inc., Canonsburg, PA) into two mesh systems with different fineness (total number of elements between 1,000,000 and 2,000,000), to detect any mesh refinement effects and to assure grid independent results. CFD simulations were performed with Fluent® (Ansys Inc., Canonsburg, PA). Steady flow simulations were performed for the peak flow condition for all mice. Note that the results from steady flow simulations have little difference from unsteady flow simulations at peak flow since the Reynolds and Wormesley numbers in mouse fetal arteries are small.

Small animal fetal blood can be considered Newtonian under physiological conditions [23]. Viscosity (*μ*), however, was assumed to depend on hematocrit (*Hct*) and calculated as follows, in accordance to studies done on 30–36 weeks human fetuses [24]:
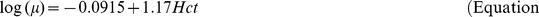
(Equation \kern 4 1)


Embryonic mouse blood at stage E10.5 was assumed to have a hematocrit of 20% [25], and was assumed to vary linearly to reach 43.8% at term [26]. Hematocrit variation over development age was assumed to be linear since this was the case in chicks [27], and in humans [28]. Using this method, viscosity of prenatal mouse blood was computed to vary from 1.87 cSt to 2.53 cSt from E11.5 to E18.5.

**Figure 2:**
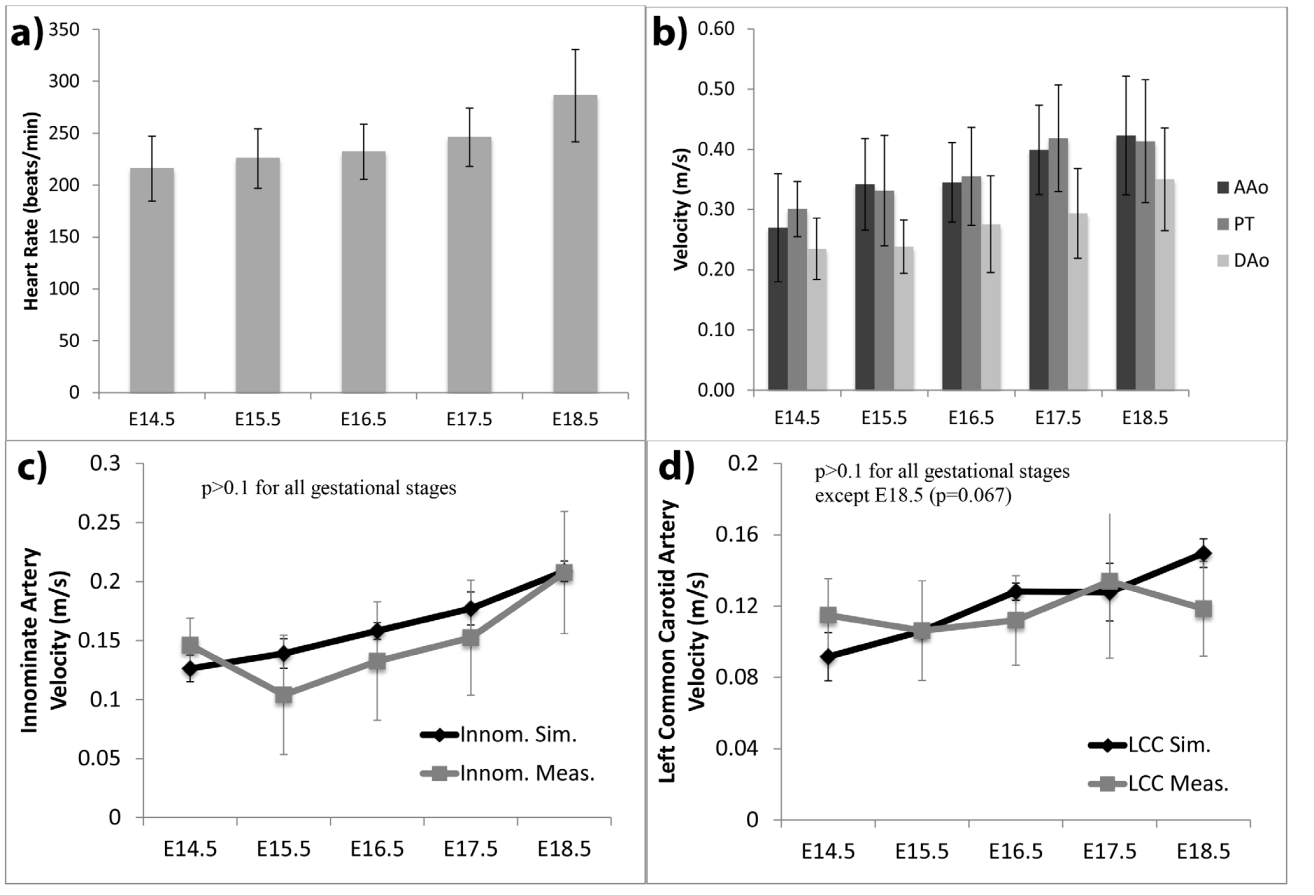
Hemodynamic measurements with high frequency ultrasound in mouse fetuses. Results of Pulsed Wave Doppler ultrasound (PW) measurements in fetal mice. a) mean heart rate across various development stages. Heart rates were positively correlated with gestational age (*ρ = *0.54, p<0.001). b) Peak flow velocities in the aortic root, pulmonary root and thoracic descending aorta, which were positively correlated with gestational age (*ρ = *0.51, 0.46 and 0.47 respectively, p<0.001 for all vessels). c–d) Plot of velocities in (c) the proximal innominate artery and (d) the left common carotid artery, comparing results obtained from the simulations and PW measurements. The simulations boundary conditions were set up with thoracic descending aorta PW measurements and Murray’s law alone, without consideration of PW measurements at the innominate and left common carotid arteries. The resulting simulated velocity data at these arteries were then observed to match PW measured ones at all gestational stages and at both arteries, indicating that the use of Murray’s law was appropriate. AAo: ascending aorta root; DAo: thoracic descending aorta; PT: pulmonary trunk root; LCC: left common carotid; Innom: innominate; Sim: simulated; Meas: measured.

Vessel walls were assumed to be rigid and impermeable with no slip boundary condition. Volumetric flow was calculated from descending aorta velocity ultrasound measurements by assuming a parabolic flow profile for use as the flow boundary condition at the descending aorta outlet. The percentage of total volumetric flow coming out of each outlet boundary was determined according to Murray’s law [29], which states that flow in vessels tend to scale according to the 3^rd^ power of vessel radius. Murray’s law is widely used in CFD studies in the literature [30]–[32] and provide physiological estimates. With the volumetric flow rate at the descending aorta outlet, we could thus calculate volumetric flow rate at other outlets, and by assuming parabolic profiles, we could compute flow velocities at these outlets. Flow velocities at the proximal innominate artery and left carotid artery outlets obtained in this manner was found to match actual ultrasound measurements at these arteries satisfactorily, as discussed in the results section, thus validating the use of Murray’s Law. In the simulations using Fluent®, all outlets were specified as “Outflow” Boundary Conditions with assumptions of zero diffusion flux for all flow variables and overall mass balance correction. Only the percentage of volumetric flow going through each outlet was specified. Pressures and velocities at outlets were not specified. Use of parabolic velocity profiles is also justified due to low Reynolds and Womerseley numbers. Flow inlet to the aorta and the pulmonary trunk artery were set as plug flow with a volumetric flow rate equal to the sum of the outlet flow rates. Velocities at the two great artery inlets were specified to be the same, according to ultrasound velocity interrogations at these two vessels (Figure 2b).

### Analysis of CFD Results

CFD data were analyzed for vessel wall shear rate (*γ*) and wall shear stress (*τ*):
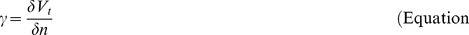
(Equation \kern 4 2)

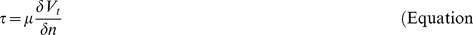
(Equation \kern 4 3)where *V_t_* is the velocity component tangent at the wall, and *n* is the normal distance from the wall surface. Wall shear stress describes the shear force per unit area experienced by vessel walls, while wall shear rate is the rate at which fluid near the vessel wall deforms. Next, the Reynolds number (*Re*) and the Womersley number (*α*) were estimated along vascular centerlines, as follows:
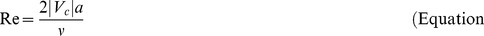
(Equation \kern 4 4)

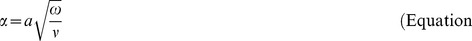
(Equation \kern 4 5)where ***V_c_*** is the velocity at a particular point on the centerline, *a* is the average of the maximum and minimum cross sectional radius at this point, *ν* is the kinematic viscosity and *ω* is the angular frequency of heart rate. Reynolds number is a gauge of the relative dominance of viscous effects and momentum effects, while Womersley number is a gauge of relative dominance between pulsatile flow effects and viscous effects The helicity of flow in the aorta was quantified using the normalized helicity parameter, calculated as the dot product of the velocity (***V***) and the vorticity (***w***), normalized by their magnitudes [33]:
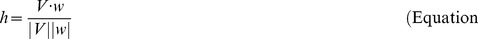
(Equation \kern 4 5)


Normalized helicity density has a range of [−1, 1]. It is a measure of whether flow at a certain point has strong vorticity and strong velocity concurrently, a condition which tends to makes fluid move in a helical motion. A value close to 0 indicates little helical flow tendency, and values close to −1 or 1 indicate significant right-handed or left-handed helical flow tendencies respectively.

### Statistical Analysis

For the comparison of simulated arterial outflow velocities at the innominate and left carotid arteries were compared with actual ultrasound measurements, the non-parametric Mann Whitney U test was applied to test whether data from the two groups were from the same population. The test was applied to all 5 gestational ages, and at both branching artery locations. Correlation and regression analyses were performed to investigate if shear stress, shear rate, and centerline velocity exhibited significant trends against vascular cross sectional areas and gestational age. For correlation analyses, Pearson’s correlation coefficient was reported, and for regression analyses, the p value for testing the hypothesis that the regression line had a slope of 0 was reported.

### Computer Models Available Upon Request

All digital models of aorta geometries, computational models and results are available upon request from the authors.

## Results

### EFIC Imaging and Vessel Segmentation

EFIC imaging provided high-resolution 3D image stacks, with sufficient intensity contrast to distinguish blood volumes from its surrounding structures such as vessel walls, myocardium, and even heart valves (Figure 1b). Figure 1c demonstrates the detailed geometry obtainable with segmentation. Figure 1d shows the representative geometry of the fetal mice great arteries at the different gestational stages included in the current study, providing a qualitative overview of the arterial growth. In order to demonstrate that EFIC can accurately capture in vivo geometries, we made measurements of the width of the thoracic descending aorta of three E19.0 mouse fetuses along the sagittal plane, using through both ultrasound and EFIC, and show that they match well: ultrasound measurement was 0.591±0.037 mm while EFIC measurement was 0.573±0.027 mm (p>>0.1).

### High Frequency Ultrasound Interrogation of Prenatal Mice

Through ultrasound imaging, we observed that wild type mouse fetus heart rate gradually increased from 216±31 beats/min at E14.5 to 286±33 at E18.5 (Figure 2a). These results fall within the range of values published by other authors. For example, we measured mean E14.5 fetus heart rate to be 215.8 beats/min, which falls between measurements of the same by Corrigan et al. (187.4 beats/min) [34] and Phoon et al. (261 beats/min) [35]. We measured ejection duration as a ratio of cardiac cycle to be a gradual decrease from 0.44 to 0.38 between E14.5 to E18.5, slightly higher but close to that of Corrigan et al (0.39 to 0.33 from E14.5 to E18.5). Peak flow velocities at various arteries are shown in Figure 2b. We noted that our measurements are close to previously published data [36]. Statistical analysis showed that velocities had a significant trend of increasing with gestational age. At any of the gestational age investigated, the velocity measured in the aortic root was the same as that measured in the pulmonary root. We used this observation to guide our specification of the boundary conditions at the aortic and pulmonary root: uniform velocity plug flow profile with the same velocity at these two boundaries. A second order polynomial curve was fitted to the plot of the descending aorta velocities versus development stages, and was found to have the following form:
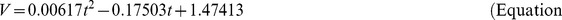
(Equation \kern 4 6)where *V* is the velocity magnitude in the descending aorta, and *t* is the gestation age in number of days. This curve was used to determine flow boundary conditions for input into the CFD simulations.

Ultrasound interrogations were further used to validate the use of Murray’s law in the simulations. The simulations were performed based solely on ultrasound measurements of the mid-thoracic descending aorta and Murray’s law. The resulting maximum velocities at the innominate artery and left common carotid artery were found to agree with PW ultrasound measurements at these locations, as shown in Figures 2c and 2d. Statistical testing demonstrated that simulated velocities were not significantly different from ultrasound-measured velocities (p>0.1 for all gestational ages, at both arterial branches, except for E18.5 at the left common carotid artery, where p = 0.067). This showed that Murray’s law approach to setting the boundary conditions was satisfactory.

### Quantitative Fetal Mouse Great Arterial Geometry

**Figure 3:**
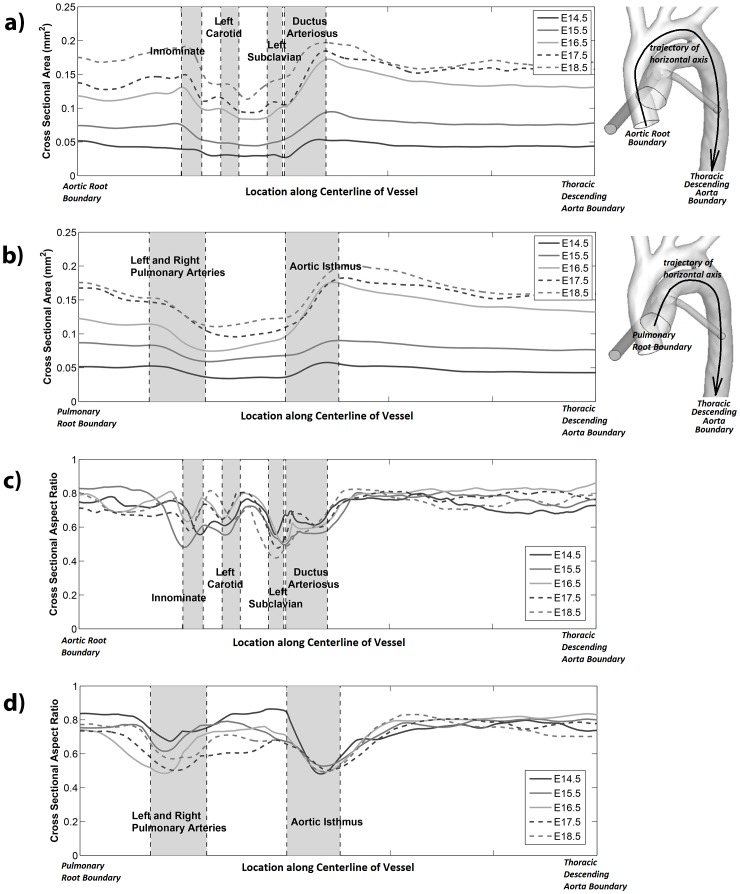
Detailed analysis of geometric parameters along the length of great arteries. These plots show how geometric parameters change from one cross section to the next, moving along the vascular centerline from the great arterial roots to the distal descending aorta. In these plots, the vertical axis denotes a geometric parameter, which is either vascular cross sectional area (a,b) or vascular cross sectional aspect ratio (c,d); and the horizontal axis denotes the location on the centerline of vessels. In plots (a) and (c), the horizontal axis denotes the centerline trajectory from the aortic root boundary to the aortic isthmus to the thoracic descending aorta boundary; while in plots (b) and (d), the horizontal axis denotes the centerline trajectory from the pulmonary root boundary to the ductus arteriosus to the thoracic descending aorta boundary. All centerline distances were scaled to fit the plot, such that locations of arterial branches align and comparisons between different development stages could be performed.

Figure 3a and 3b show the lumen cross-sectional area of the aorta, the pulmonary trunk and the *ductus arteriosus*, plotted as a function of centerline distance from the aortic root. Centerline distances were scaled in such a way that the locations of major vascular junction from different animals aligned with one another. This way, variables from different animals of the same age can be averaged, and changes in averaged variables over ages can be observed. For all gestational stages, the ascending aortas appeared larger than the aortic arch, but approximately the same size as the descending aortas. The pulmonary trunk was approximately the same size as the descending aorta during E14.5 and E15.5, but during the later stages, appeared slightly smaller. For all arteries studied, there was a gradual increase in vascular lumen cross sectional area over developmental age.

Figure 3c and 3d show the cross sectional aspect ratio (minimum diameter dimension divided by maximum diameter dimension) of the aorta, the pulmonary trunk and the *ductus arteriosus*. The aspect ratio is a parameter to demonstrate whether the vascular cross section is round (aspect ratio close to 1) or if it is elongated (aspect ratio closer to 0). The aspect ratio of all great arteries was approximately 0.8 in most locations. However, proximal to any arterial branch point, including the head and neck arterial branches from the aorta and the pulmonary arterial branches from the pulmonary trunk, the aspect radio decreased, indicating an elongation in the cross section. Close inspection revealed that the elongation were always in the direction of the branching.

**Figure 4:**
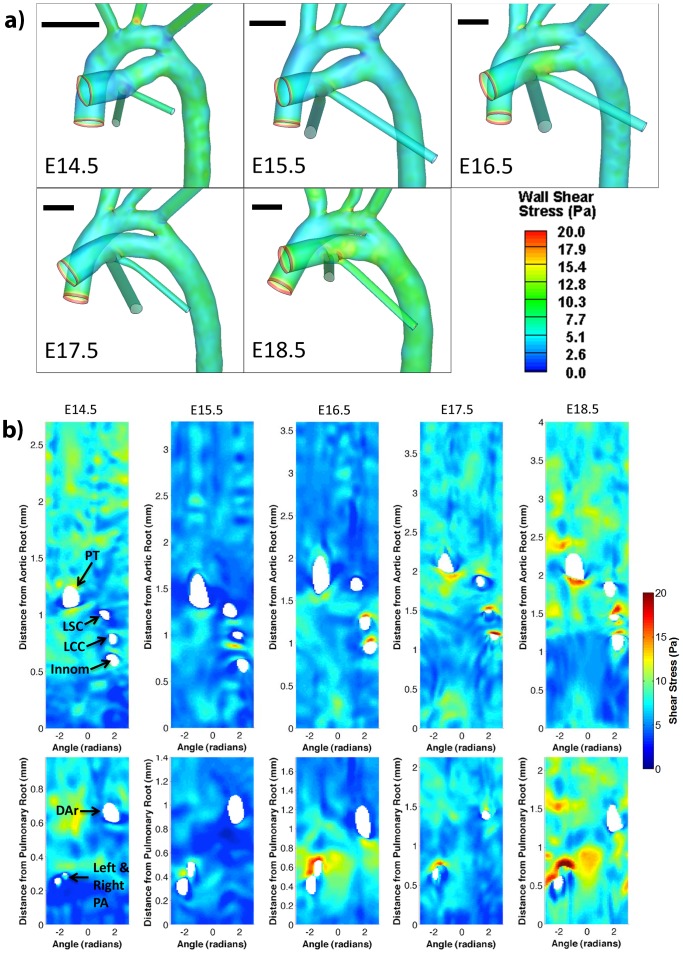
Mouse great arterial wall shear stress. Typical peak-flow wall shear stress contour diagrams of great arteries, (a) plotted on actual vascular geometries, and (b) plotted as in an “unwrapped” manner as shear stress maps: contours are plotted as a function of distance from the aortic root or pulmonary root (vertical axis) and circumferential angle (horizontal axis). Black bars in each panel in (a) denotes 500 microns. In (b), the top row are for the aorta (ascending and descending), while the bottom row are for the pulmonary trunk – *ductus arteriosus*. The right half of each map corresponds to the superior surface of the aortic arch and pulmonary trunk, or the dorsal surface of the descending aorta, and vice-versa for the left half. The holes in the map indicate locations of artery branches. PT: pulmonary trunk; DAr: ductus arteriosus; LSC: left subclavian; LCC: left common carotid; Innom: innominate; PA: Pulmonary artery.

Another notable feature was that the angle between the *ductus arteriosus* and the descending aorta was large during E14.5 (closer to 90 degrees) but becomes smaller with gestation age (closer to parallel, 180 degrees), as can be observed in Figures 1c and 4a.

### Wall Shear Results

Figure 4 shows the wall shear stress contours from the simulation results for the various development stages. In Figures 4b, shear contours were plotted as a function of distance from the aortic/pulmonary root (vertical axis) and azimuthal angle (horizontal axis). Shear stress data were shown for the peak flow time point. However, since the mouse embryonic/fetal flows had very low Reynolds (<30) and Womersley numbers (<0.5), the spatial pattern of shear stress and shear rate, after normalization by the instantaneous flow rate, did not change much over the entire duration of systole. Thus the peak flow shear spatial pattern was representative of the spatial pattern over the entire systole.

**Figure 5:**
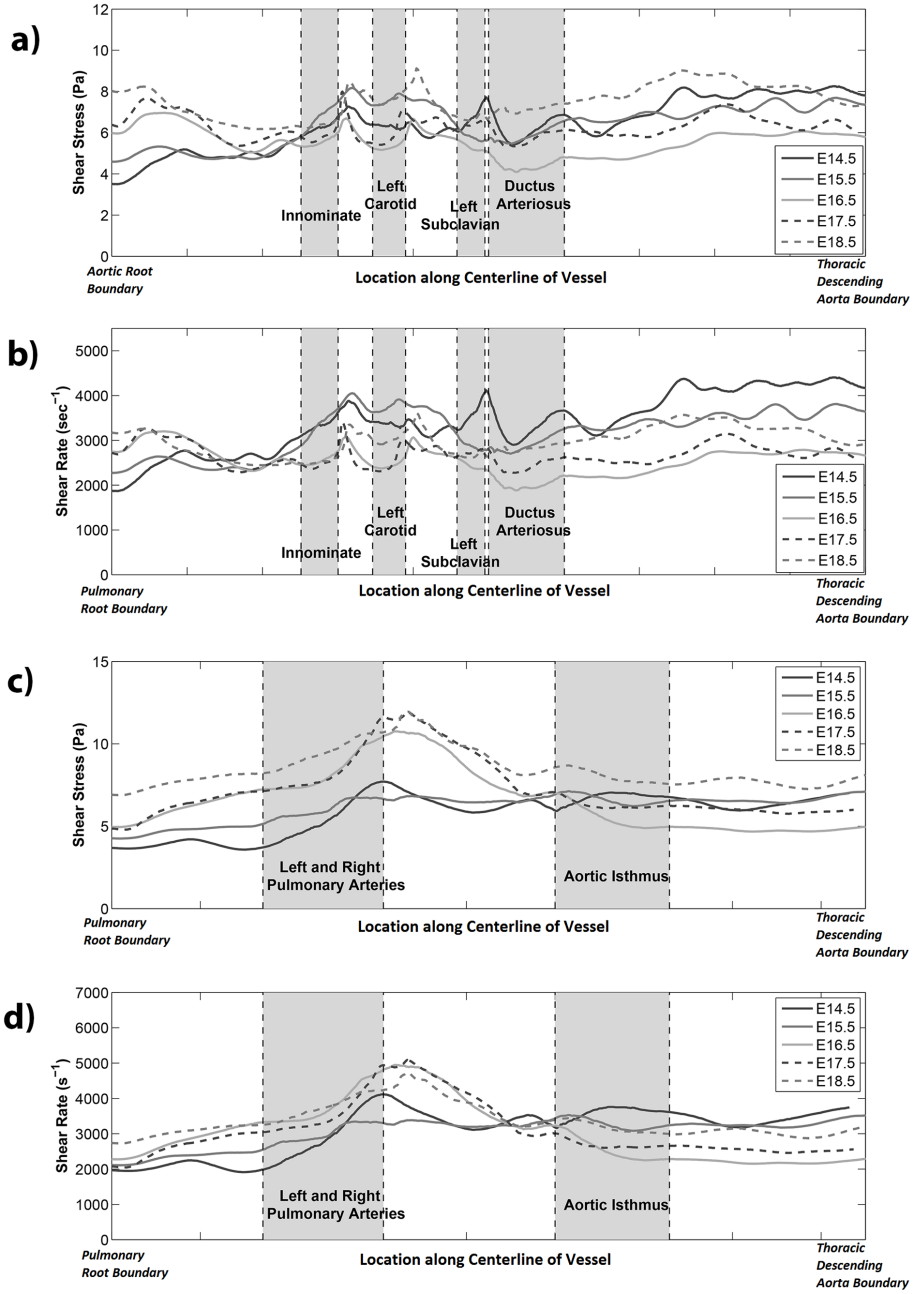
Mouse great arterial wall shear stress variation along the length of the arteries. These plots show how wall shear parameters change from one cross section to the next, moving along the vascular centerline from the great arterial roots to the distal descending aorta. The vertical axis denotes a wall shear parameter, which is either wall shear stress (a, c) or wall shear rate (b, d); and the horizontal axis denotes the location on the centerline of vessels. Wall shear parameters are averaged over the entire cross section at each vascular centerline location. In plots (a) and (b), the horizontal axis denotes the centerline trajectory from the aortic root boundary to the aortic isthmus to the thoracic descending aorta boundary; while in plots (c) and (d), the horizontal axis denotes the centerline trajectory from the pulmonary root boundary to the ductus arteriosus to the thoracic descending aorta boundary. Centerline distance from the aortic/pulmonary roots were scaled such that the geometric landmarks such as the locations of arterial branches could be plotted at the same location across different ages to allow comparisons of features across different gestation stages.

Shear stress and shear rate were fairly uniform over the great arterial walls. Spatial variation in shear appeared to be related to vessel branching. For example, on the aorta, it was observed that shear stress and shear rate were elevated distal to branching head and neck arteries and proximal to the *ductus arteriosus* branching point. Similarly, on the pulmonary trunk – *ductus arteriosus* junction, shear stress and shear rate increased distal to the left and right pulmonary artery branching point. The elevation of shear distal to arterial branches appeared to be more significant with increasing gestation age. Further, lateral to some of the branching arteries, the aortic wall appeared to have reduced wall shear stresses. The plots of hemodynamic shear versus distance from the aortic root or pulmonary root are shown in Figure 5 for all the developmental ages studied. The elevation of shear stresses distal to arterial branches on the aorta described above could be observed in these plots as magnitude spikes distal to branching locations.

### Variation of Geometric and Hemodynamic Shear with Gestational Age

**Figure 6:**
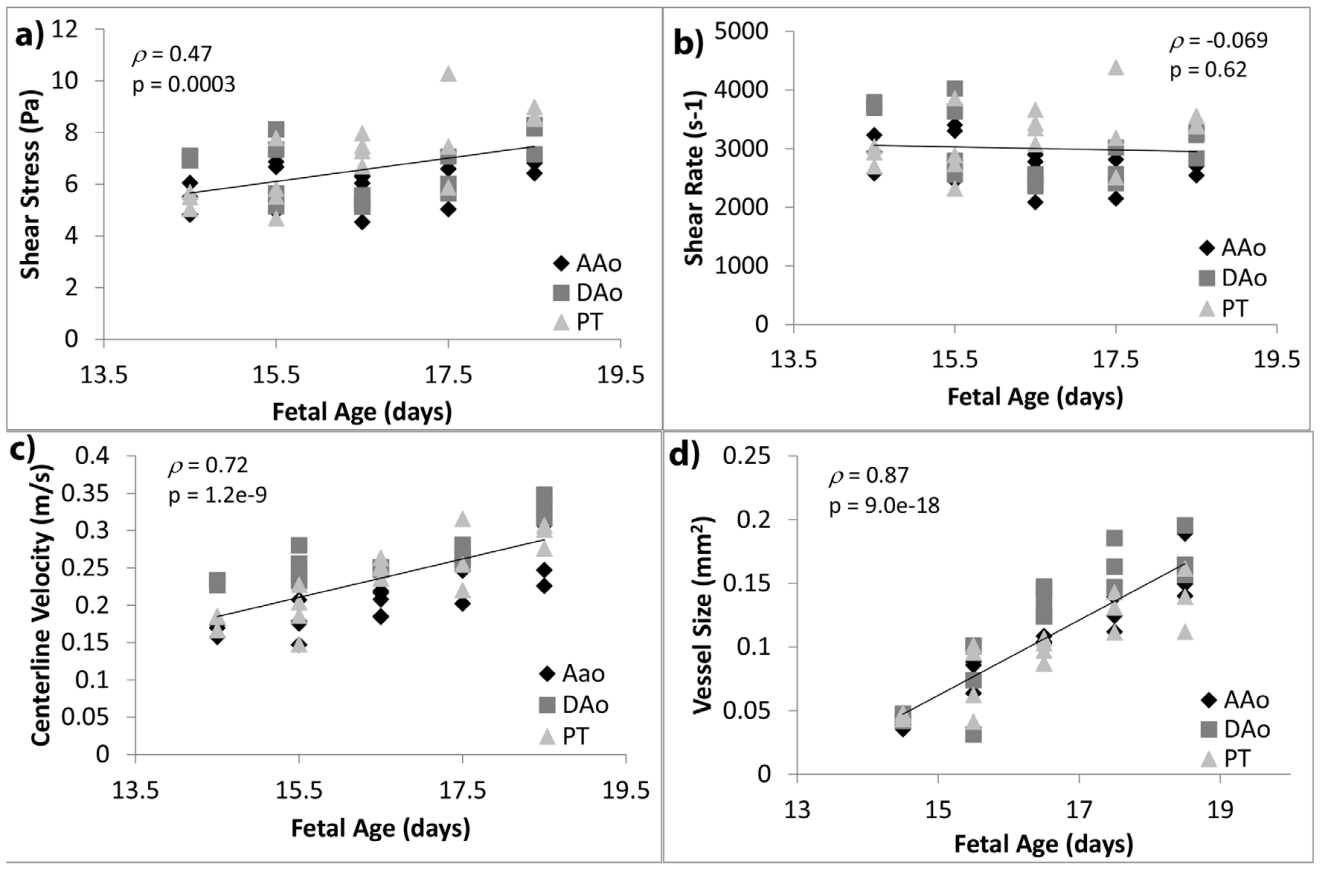
Variation of geometric and mechanics parameters over gestational ages. (a) Wall shear stress, (b) wall shear rate, (c) centerline velocity, and (d) vessel size averaged within specific arteries and plotted against developmental age. Correlation and regression analysis were performed for data for all three vessels cumulatively. AAo: ascending aorta; DAo: descending aorta; PT: pulmonary trunk and the *ductus arteriosus*.

Figure 6 plots wall shear stresses, wall shear rates, centerline velocities, and vascular cross sectional areas versus gestational ages. These parameters correspond to values at peak flow time point, and were averaged for specific arteries: the ascending aorta is defined as the aorta proximal to the aortic isthmus; the descending aorta is defined as the aorta distal to the isthmus; the pulmonary trunk is defined as the main pulmonary artery proximal to the left and right pulmonary artery branch points; and the ductus arteriosus is defined as the connection between the pulmonary trunk and the descending aorta. The pulmonary trunk and ductus arteriosus are analyzed collectively as combined data, since flow into the left and right pulmonary arteries are low during fetal stages, and most flow from the pulmonary trunk drains into the *ductus arteriosus* as if they were a continuous vessel. In each figure, statistical analyses were performed cumulatively with data from all vessels. Shear stress was observed to have a positive trend with age (*ρ = *0.47), but shear rate was found to be independent of age (*ρ* = −0.07; *p* = 0.62). Centerline velocities and vascular sizes were observed to have highly significant increasing trends with age (*ρ = *0.72 and 0.89 respectively), which were much more significant than that of shear stress.

We noted that due to the low Reynolds number, the velocity at the centerline of the vessel is close in value to the maximum velocity within the same cross section. Thus the centerline velocity should be just as good as the maximum velocity as a good guide for the amount of flow within the vessel.

### Variation of Geometric and Hemodynamic Shear with Vascular Sizes

**Figure 7:**
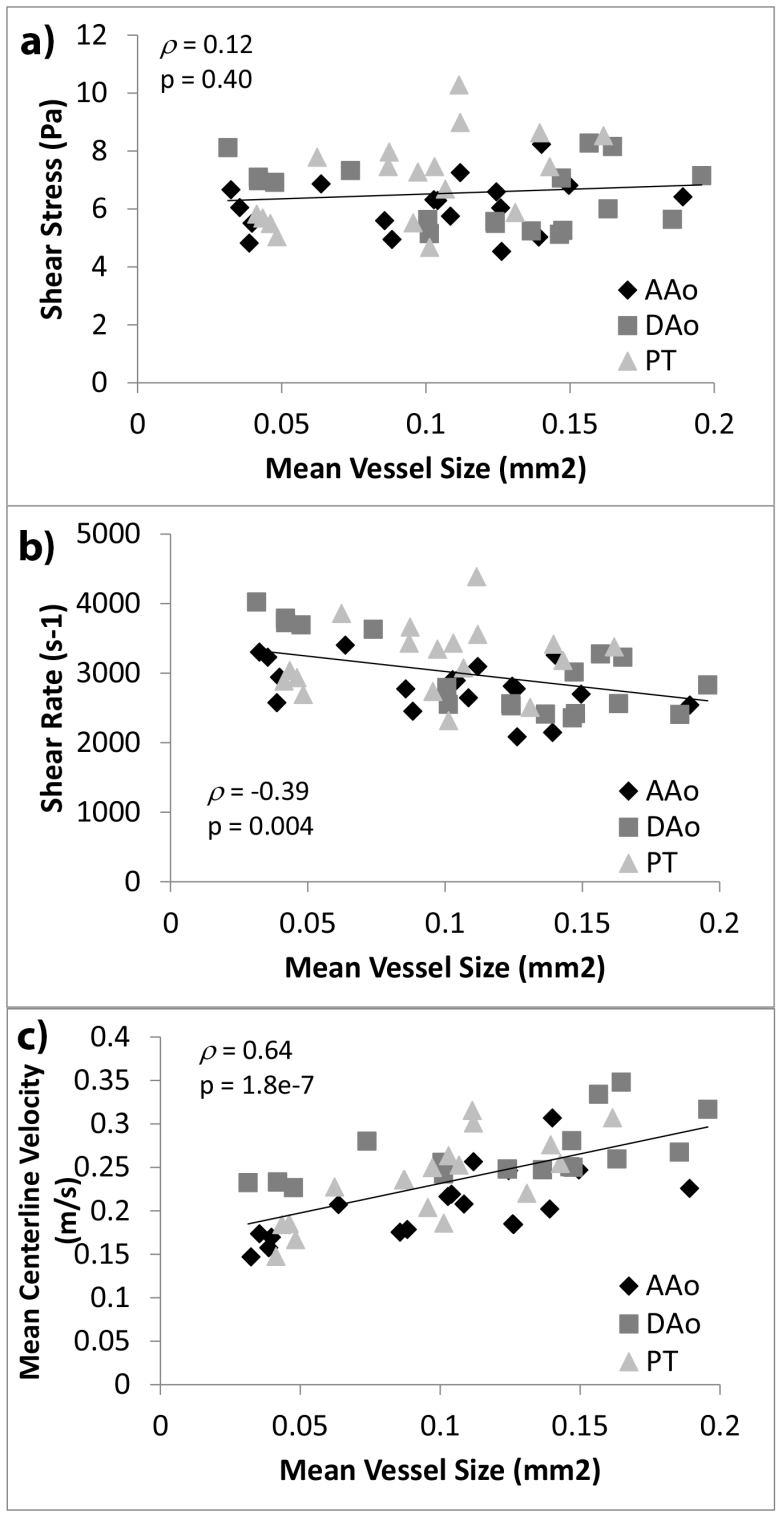
Variation of geometric and mechanics parameters across different vessel sizes. (a) Wall shear stress, (b) wall shear rate, and (c) centerline velocity averaged for specific vessels, plotted against average cross sectional area of the same vessel. Data for all ages between E14.5 and E18.5 were plotted. Correlation and regression analysis were performed for data for all three vessels cumulatively. AAo: ascending aorta; DAo: descending aorta; PT: pulmonary trunk and the *ductus arteriosus*.

**Figure 8:**
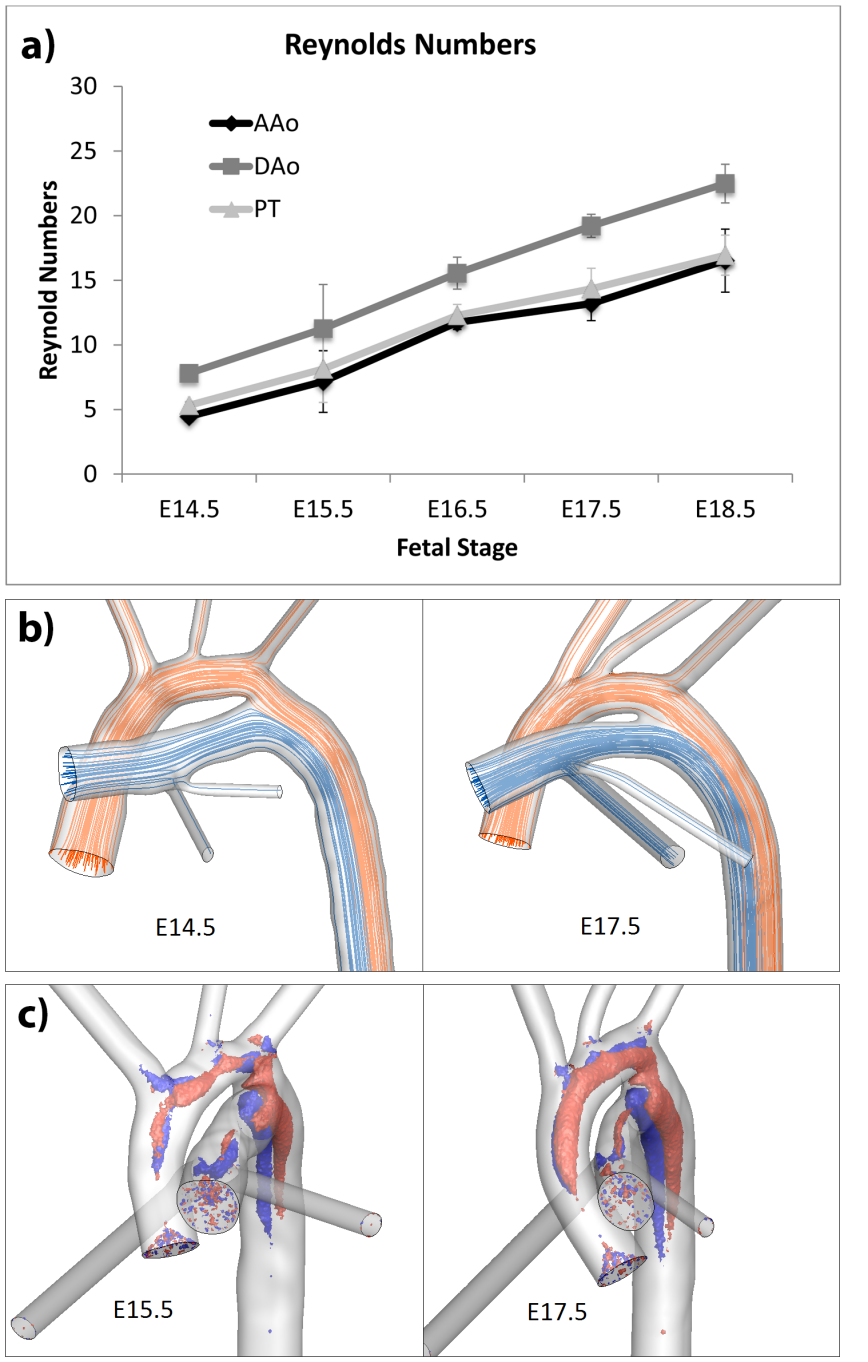
Fluid mechanics parameters of flow in mouse great arteries. a) Reynolds numbers of flow in specific arteries, averaged along the entire arterial centerline, at different developmental stages. DAo: descending aorta; AAo: ascending aorta and aortic arch; PT: pulmonary trunk including the *ductus arteriosus*. b) Peak flow streamlines in the great arteries of embryonic mouse at E14.5 (left) and fetal mice at E17.5 (right). c) Normalized helicity iso-surfaces in the flow in great arteries of embryonic and fetal mice of various developmental stages, showing the development of classical Dean flow in the great arteries. There was a general increase helicity over age, and equal intensity in right-handed (red, normalized helicity = 0.2) and left-handed (blue, normalized helicity = −0.2) helical structures.

Figure 7 plots wall shear stresses, wall shear rates, and centerline velocities against vascular cross sectional areas, in the same manner as presented by Figure 8. It was observed that shear stresses were statistically independent of vascular sizes (*ρ = *0.12; *p* = 0.40), shear rates have a negative trend with vascular sizes (*ρ* = −0.39), and centerline velocities exhibited a much more significant trend with vascular size (*ρ = *0.64).

### Flow Streamlines in Embryonic and Fetal Great Arteries

Centerline Reynolds numbers were averaged throughout the entire length of the arteries and plotted in Figure 8a, showing that the peak flow Reynolds numbers in the fetal great arteries were very low, being less than 30. In contrast, peak flow Reynolds number in adult mice is between 300 and 400. With increasing developmental age, the increase in flow velocities and vessel dimensions led to a linear, gradual increase in the Reynolds numbers.

Investigation of the flow streamlines results showed that flow was very orderly, as expected of low Reynolds number flows (Figure 8b). Even at the confluent of the aortic arch and the *ductus arteriosus*, there was little fluid mixing and unsteadiness as fluid from the two contributing arteries came together. The lack of fluid mixing from the two contributing arteries continued even far downstream of the *ductus* insertion point in the descending aorta.

### Flow Helicity in Embryonic and Fetal Great Arteries

Helicity iso-surfaces were shown in Figure 8c for arteries of various developmental stages. Weak double-helical secondary flow structures appeared in the aortic arch and the proximal descending aorta as shown in the figure. Helicity in both right-handed and left-handed directions were approximately of the same intensity, and general helicity intensity was observed to increase with developmental ages.

## Discussion

In the current study, we established a practical pipeline to study the fluid mechanics in the great vessels of fetal mice. We showed that this method can elucidate details of geometric features and fluid mechanics loading. Further, we found that while vascular dimensions and flow velocities demonstrated highly significant increasing trends with developmental age; vascular wall shear stress and wall shear rate demonstrated weak or no trends; and while flow velocities had strong increasing trends against vascular sizes, wall shear stresses and wall shear rates had weak trend or no trends. This observed relative constancy of the shear environment may suggest the existence of a homeostatic regulatory mechanism.

### The EFIC-UBM-CFD Method

CFD simulation is a well-established method to study cardiovascular fluid mechanics [37]–[39]. In the current study, we adapted two recent technologies, EFIC and UBM, to aid CFD simulations, establishing a new methodology for analyzing fetal mice arterial fluid mechanics. EFIC has been used previously for detailed imaging of mouse anatomy for embryology studies and phenotyping mutant mice [16], [17], [40]. The current study taps into its ability to obtain high-resolution 3D geometry for fluid mechanics studies.

Cryo-EFIC allows the snap-freezing of fetal samples quickly after harvest to minimize dimension change artifacts resulting from specimen handling, and is a quick and easy (automated) means of obtaining high-resolution geometries of small samples. The drawback of EFIC for fetal mouse fluid mechanics studies, however, is that EFIC can only provide static geometries, and cannot provide descriptions of *in vivo* cardiovascular motions. Furthermore, EFIC cuts and destroys the sample, unless a system to collect cut slices from the cryo-microtome is designed and installed. Nonetheless, we show that Cryo-EFIC is a feasible alternative to existing established methods such as resin casting [41] or micro-CT [42].

The inclusion of UBM is essential in our overall methodology, due to its ability to measure in vivo velocities in small animal fetuses such as the mouse, which is crucial to our specification of boundary conditions. With current technology, UBM is arguably the only method capable of obtaining this measurement: Magnetic Resonance Imaging requires synchronization with fetal heart rate, which remains a challenge; and Optical Coherence Tomography requires optical access, which is not available in the mouse. The relatively recent introduction of high frequency ultrasound in small animal research [43] has thus enabled our study.

### Variation of Hemodynamic Shear in Embryonic and Fetal Great Vessels with Age and Vascular Size

The main finding of the current study is that wall shear stresses and wall shear rates had a weak trend with developmental age and vascular sizes, or no statistically significant trends at all; this is unlike centerline flow velocities, which had strong correlations with developmental age and vascular sizes, and unlike vascular sizes, which had strong correlation with developmental age. In particular, shear stresses were statistically independent of vascular sizes, while shear rates were statistically independent of developmental age. Thus while these prenatal vessels grew in sizes and hosted increasing flows, the hemodynamic shear environment of the vessel walls were relatively constant.

We speculate that this relative constancy of the shear environment was due to the regulation of great arterial growth by the fluid shear, where vessel walls were capable of sensing shear, and relied on shear stimuli to control growth rate of the vessel. In this manner, the blood vessels were able to grow at the appropriate rate that corresponds to circulatory requirements of the body. Further studies, however, are needed for verifying this hypothesis.

The notion that prenatal vascular walls are capable of sensing shear is a subject of current investigation [5], [44], [45]. In vitro studies on cell layers showed alterations in expression levels of shear-sensitive factors and genes when hemodynamic shear was altered [5], [45], and some authors attributed the underdevelopment of the left heart and aorta in hypoplastic left heart syndrome patients to the aortic valve atresia [46], which could have prevented flow stimuli.

In adult arteries, it is well known that chronic exposure to altered shear stress causes vascular remodeling (widening or narrowing) to accommodate the change and restore the shear stresses to the initial levels [47]. Biological mechanisms for this response has been reported [48], and several cellular components of endothelial cells were implicated to be shear sensor, including integrins, cell junctions, ion channels, and the glycoaclyx [48], [49]. We hypothesize that embryonic and fetal arteries possess a similar response mechanism to maintain hemodynamic shear homeostasis, and that this mechanism informs the pre-natal great arteries on how fast to grow to keep in pace with circulatory requirements of the body.

If the endothelium senses fluid shear through shear-force-induced deformation of endothelial cells, leading to conformational changes of structural proteins, the study of shear stress is more relevant than shear rate. On the other hand, if endothelial cell relies on cell surface protrusions to sense shear, the study of shear rate becomes more relevant, because the amount of deformation surface protrusions experience depends on both viscosity of fluid and velocity of flow moving past it, and velocity is directly related to shear rate due to its definition (shear rate is defined as the velocity gradient at the wall). If the endothelial cell relies on stresses in the arterial wall to sense shear, however, the converse will be true, arterial wall stresses will depend on how much force is imparted to it by flow, which is directly dependent on the magnitude of shear stress. In the current study, we presented results of both wall shear rate and wall shear stress, due to the uncertainty of which endothelial shear sensing mechanism is most relevant. We noted that for embryonic and fetal blood, viscosity can change substantially with development due to the increase in hematocrit, and thus shear rate and shear stress values will not be equivalent across developmental stages. We observed that wall shear rate appeared to stay more constant over developmental ages than shear stress. This may indicate that surface protrusion mechno-sensors play a dominant role.

Recent investigation has identified primary cilia as possible mechanotransduction organelles in endothelial cells [9]. Primary cilia are found in the pre-natal heart and arteries where low shear stresses are expected, but are absent where high shear stresses are expected, most likely due to cilia dissociated under a high shear environment [45]. Our results indicate that mouse great arteries experienced much shear stresses about an order of magnitude higher than those used by investigators of cilia mechanobiology [9], [50], and at these levels of shear stress, endothelial cilia would have disassemble, according to a previous study [51]. Consequently, it is unlikely that primary cilia play a role in sensing hemodynamic shear to regulate vessel growth during later embryonic stages and during fetal stages. In humans, shear stresses were estimated to be lower than in mouse [52], and it is possible for cilia to remain assembled on human endothelium. This mechanism of shear sensing, if it exists in humans, would most likely not be conserved in small animals.

### Spatial Variation of Hemodynamic Shear

Our results indicated that the most significant spatial variations in shear stresses were related to arterial branching. Shear magnitudes elevated distal to arterial branches and were reduced lateral to the branches, and were related to the fluid mechanics of fluid branching [53]. The elevation of shear stress on the aortic wall directly proximal to the ductus arteriosus insertion point was related to sharp local curvature of vessel wall. Other than these variations, shear magnitudes were fairly uniform across the surfaces of the great arteries, and only minor variations due to geometric unevenness were observed. This spatial uniformity in shear stresses was most likely related to the low Reynolds number of mouse embryonic/fetal great arterial flow, where flow was highly ordered and little secondary flow and fluid mixing occurred.

The spatial heterogeneity of wall shear stresses observed in the current study could indicate spatially varied biological responses is being elicited in the great vasculature, which could play a role in the remodeling of the vasculature for maintenance of function, such as the geometry of arterial branches that transport efficiency.

The magnitude and spatial pattern of shear stresses elucidated in the current study will be helpful to future investigation of fetal vascular mechanobiology using mouse tissues, and for identifying the mechanism of endothelial shear sensing, and will be helpful especially in experiments using mouse models.

### Great Arterial Geometry Features

From the geometric data, fetal mouse aortic arches appeared adapted for energy efficiency at the branching point. This can be observed from the way the aortic arch elongated at branching locations towards the branching artery, which could be taken as a geometric adaption of the vasculature to channel blood flow into the branching artery in an energy-efficient manner, where sharp turns in fluid flow are avoided. In the same manner, the decrease of the insertion angle with age of the *ductus arteriosus* as it inserts into the descending aorta could be a remodeling adaptation to improve energy efficiency of fluid draining from the pulmonary trunk/*ductus arteriosus* into the descending aorta.

### Flow Streamline Patterns

The orderly flow streamlines and the lack of flow separation despite large turning angles can be explained by the low Reynolds numbers found in the fetal mouse great arteries investigated, which indicated that viscous forces dominated the flow. Low Reynolds numbers also explained the minimal mixing of flow even at the confluence of *ductus arteriosus* and aortic arch flow in the proximal descending aorta (Figure 8b).

Consequence of the highly viscous environment, secondary flows were weak, as they were more quickly dissipated than they were generated. The observed secondary flows observed were Dean-like [54], [55] in nature, manifesting as toroidal or double-helical flow structures in the ascending aorta and the aortic arch. This was a result of centrifugal forces of fluid in the curved arteries, which moved fluid in the center towards the outer wall of the curvature, and in the process, pushing fluid near the outer wall to flow along the walls towards the inner wall of the curvature. Double helical flow patterns are also observed in rabbit [31] and human [33] aortas.

## Conclusions

In the current study, we have developed a method to study fluid mechanics in vessels of embryonic and fetal mouse arteries at late stage of development, using a combination of EFIC, UBM and CFD, and demonstrated its ability to reveal detailed geometric, kinematic and fluid mechanics information. We found that wall shear stresses and wall shear rates had weak or no trends with gestational age and vascular sizes, even though vascular sizes and flow velocities had strong increasing trends with age, flow velocities had strong increasing trends with vascular sizes. This observed relative constancy in shear environment may indicate that vascular growth is regulated by a hemodynamic shear sensing mechanism, which sought to maintain shear at the same level by growing vessel dimension with the increase in flow demanded by the body.

## Limitations

Our computations were performed assuming non-mobile rigid walls, which does not capture diastole-systole vascular distension. Using ultrasound, we measured that this distension to be approximately 13% of diastolic vascular dimension. However, our computational results can serve as a time-averaged approximation to in vivo shear environments, and several previous work has reported that small wall motions have minor effects on time-averaged wall shear stress computations [56], [57].
